# The impact of time of neutering on weight gain and energy intake in female kittens

**DOI:** 10.1017/jns.2017.20

**Published:** 2017-05-15

**Authors:** David Allaway, Matthew Gilham, Alison Colyer, Penelope J. Morris

**Affiliations:** WALTHAM Centre for Pet Nutrition, Melton Mowbray, Leicestershire, UK

**Keywords:** Neutering, Cats, Energy intake, Weight gain, Behaviour, Appetite, Obesity, BCS, body condition score, BW, body weight, CN, conventional neutering, EN, early neutering, TER, total energy requirements

## Abstract

Neutering is a risk factor for obesity in companion animals. In a study to determine the total energy requirements of kittens (15–52 weeks) the impact of neutering and age when neutered on intake and body weight (BW) was investigated. Females (*n* 14), neutered when 19 (early neuter; EN) or 31 (conventional neuter; CN) weeks old (*n* 7/group), were individually fed to maintain an ideal body condition score (BCS). EN kittens gained weight gradually whilst CN kittens’ BW gain slowed from week 24, weighing less than EN kittens from week 30 with a reduced energy intake (kcal/kg BW^0·67^) in weeks 24–32 (*P* < 0·05). Following neutering, CN cats’ BW and energy intake increased rapidly (energy intake CN > EN in weeks 36–40). Although EN required earlier diet restriction, acute hyperphagia and increased rate of BW gain following neutering were not observed. Earlier neutering may aid healthy weight management through growth when regulating intake to maintain an ideal BCS.

Excess weight is a major health concern in cats^(^^1^^)^, with studies suggesting that up to 52 % of the adult cat population are overweight or obese ^(^^2^^–^^5^^)^. Overweight is associated with an increased risk of mortality, predisposes individuals to chronic metabolic disorders, such as diabetes mellitus and hepatic lipidosis, and reduces quality of life through chronic diseases, such as osteoarthritis^(^^6^^)^. Owners seeking advice to support weight management of their pets need clear and accurate advice, as well as access to products that meet all nutritional requirements at the appropriate energy intake. The current National Research Council (NRC) guidelines calculate that energy requirements for adult cats are 100 kcal/kg BW^0·67^ (418 kJ/kg BW^0·67^) for normal-weight cats and 130 kcal/kg BW^0·4^ (544 kJ/kg BW^0·4^) for heavy cats^(^^7^^)^. However, feeding to the current 2006 NRC guidelines may be inappropriate, as a meta-analysis of publications (reported in English and published from 1933 to 2009) indicated that feeding guidelines should be reduced to 77·6 kcal/kg BW^0·71^ (324·7 kJ/kg BW^0·71^)^(^^8^^)^.

Whilst a healthy, stable body weight (BW) may be manageable in adult cats by feeding nutritionally complete diets near to this intake, adjusted by owners to their individual cat requirements by monitoring the body condition score (BCS), owners may also seek advice on an age-appropriate intake through growth and sexual development. This is especially important, as evidence indicates that risk factors for being overweight as an adult include the rate of growth in *ad libitum*-fed cats^(^^5^^)^, feeding a dry diet and restricted exercise at age 12·5–13 months^(^^9^^)^. Furthermore, neutering, often carried out in the first year of life, is also a major significant risk factor for obesity. Studies report that cats have acute changes in intake post-neuter when fed *ad libitum*, gaining BW and fat mass within 8–12 weeks of neutering^(^^10^^,^^11^^)^ that may persist throughout the individual's adult life^(^^12^^)^. As neutering is often undertaken in the first 7 months, awareness of normal growth and development may be challenging for owners wishing to manage healthy weight in the post-neuter phase.

To offer options to prevent neuter-associated weight gain during growth it is useful to gain an understanding of the different factors that may underpin the post-neuter dysregulation of self-regulated food intake. As oestrogen can reduce dietary energy intake^(^^13^^,^^14^^)^, the level of sexual maturity at neutering may be particularly important. A study undertaken to assess the total energy requirements (TER) of kittens (15–52 weeks) was used to investigate the impact of neutering and age when neutered on intake and BW. The data from the female kittens are reported and discussed here.

## Materials and methods

### Animal maintenance and diets

A total of fourteen female kittens were recruited from fourteen litters when aged between 14 and 17 weeks of age to a trial feeding a single batch of a nutritionally complete^(^^7^^)^, commercial dry diet formulated to support kittens through growth (Royal Canin Kitten) to 1 year of age (confirmed nutritional composition: moisture 6·1 %, protein 33·4 g, fat 19·2 g, ash 6·9 g, non-fermentable extract 33 g, predicted metabolisable energy 1707·9 kJ/100 g as fed). All kittens were housed in purpose-built, environmentally enriched housing at the WALTHAM Centre for Pet Nutrition, in accordance with the centre's research ethics policy and UK Home Office Regulations and cared for according to WALTHAM kitten guidelines. Kittens were individually fed to maintain an ideal BCS (based on the Size, Health And Physical Evaluation (S.H.A.P.E.™) seven-point scale^(^^15^^)^) and evaluated by a group of trained panellists, with weekly assessments to determine whether any changes in intake were required. Cats had free access to fresh drinking water.

### Study design

The kittens were housed together in a single social group, and allocated to one of two groups (seven kittens in each), based on the age at which they were to be neutered. Neutering was performed as part of normal veterinary practice at WALTHAM and occurred at one of two time points, one defined as early (early neutering; EN), at 19 weeks of age and the other, defined as conventional (conventional neutering; CN), at 31 weeks of age. Whilst neutering may occur earlier (6–12 weeks of age), 19 weeks is consistent with a previous study^(^^11^^)^, where cats were fed a dry diet *ad libitum*. The choice of 31 weeks as a later time point is not a specific ‘conventional’ time but is consistent with neutering following sexual development and was primarily to allow an entire control group for the longest time within current husbandry practice at WALTHAM. One kitten was removed from this study (EN group) at 20 weeks of age for health-associated reasons unrelated to the study.

Food intake (g) was measured after each meal and calculated (amount offered – refused) on a daily basis, and BW (kg) and BCS (seven-point scale) weekly. Spontaneous physical activity levels were assessed (average count for 24 h periods over three consecutive days) using Actical devices (Philips Respironics) attached to the cats’ collars, when cats were 19, 25, 31, 37, 43 and 52 weeks of age.

To ensure kittens were allowed to grow normally, they were offered a ration to ensure excess was offered, with the weekly food ration increased by 10 % if more than 90 % was consumed in any meal in the previous week. A proviso was that the ration was restricted if the kitten was considered to be at risk of going above an ideal BCS, measured weekly. When required, diet intake was reduced in 10 % increments with weekly review by the trained BCS panellists to assess progress, so in practice each cat was managed on an individual basis.

### Statistical analysis

A linear mixed-effects model was used to analyse the daily energy intake (kcal/kg BW^0·67^) and BW (kg) with neuter group, age (in weeks) and their interaction as fixed effects and cat as a random effect. Planned contrasts were performed between EN and CN groups at each week. A family-wise statistical test level of 0·05 was used and estimates are reported as means with 95 % family-wise CI. Statistical analysis was performed using R version 3.2.4^(^^16^^)^.

## Results

**Fig. 1.**
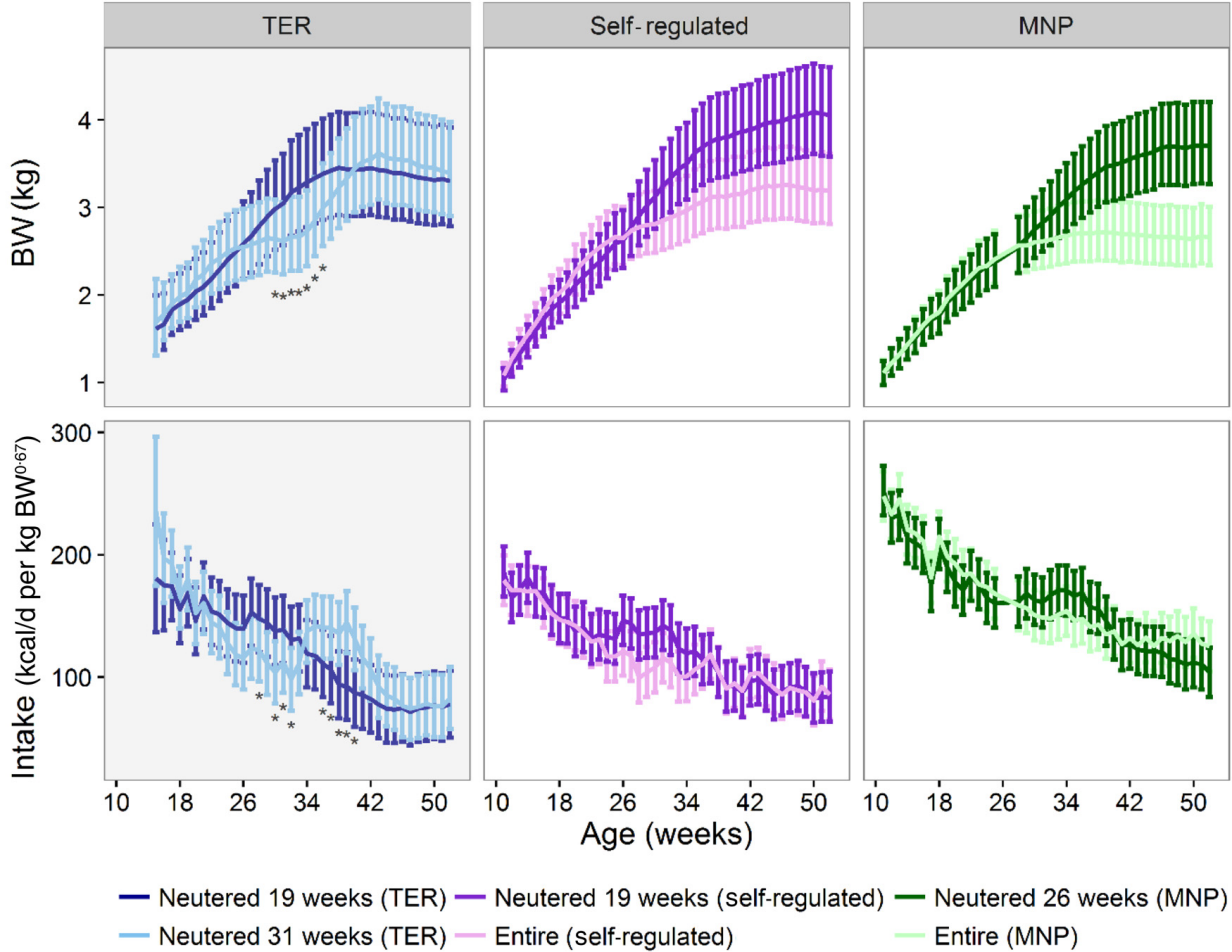
Upper row: Weekly body weight (BW; kg) of female kittens (mean and 95 % CI). The total energy requirement (TER) study reported here (female cats neutered at 19 and 31 weeks) in the left-hand panel is compared with data from two previous trials that were carried out at the WALTHAM Centre for Pet Nutrition at different times and with different dietary management regimens. The ‘self-regulated’ trial^(^^11^^)^ (middle column) describes data from a study where the intake and weight data were obtained from female cats fed a commercial dry diet *ad libitum* and where cats neutered at 19 weeks of age were compared with a group that remained entire to 1 year of age. The macronutrient profile (MNP) food selection trial (right-hand column; AK Hewson-Hughes, VL Hewson-Hughes, R Staunton and SJ Simpson, Raubenheimer D, unpublished results) measured daily intakes from female cats offered excess of each of three wet diets differing in macronutrient composition at each meal, enabling them to select a macronutrient composition and eat *ad libitum*, where the neutered group was neutered at 26 weeks of age and compared with an entire group to 1 year of age. Lower row: Daily energy intake (kcal/d per kg BW^0·67^) for the females of each of the three studies (mean and 95 % CI). Note the rapid increase in intake post-neuter in cats neutered at 31 weeks of age compared with at 19 and 26 weeks of age (left, middle and right-hand panels, respectively). * For the TER study, significant difference within the pane between the two groups (adjusted *P* < 0·05) at that time point. To calculate energy in kJ, multiply kcal by 4·184.

EN kittens gained weight gradually to 38 weeks of age (Fig. 1, TER; left-hand column) but all needed dietary management at some stage. CN kittens’ BW gain slowed from week 24, they weighed less from week 30 (*P* < 0·05) and had a lower energy intake (kcal/kg BW^0·67^; *P* < 0·05) between weeks 24 and 32 (significant in weeks 28, 30–32, *P* < 0·05; up to 32 (95 % CI 1·8, 61·8) kcal/kg BW^0·67^ (134 (95 % CI 7·5, 258·6) kJ/kg BW^0·67^) different in week 28). Following neutering (week 31), with adjustments in accordance with feeding protocol, both the intake and BW increased rapidly (significantly so in intake compared with EN between weeks 36 and 40, *P* < 0·05; up to 51 (95 % CI (20·7, 80·6) kcal/kg BW^0·67^ (213 (95 % CI (86·6, 337·2) kJ/kg BW^0·67^) at week 38). Of the thirteen kittens, eleven had a BCS that required intake restriction at some stage (with two of the EN group requiring restriction pre-neuter) and all cats in the study had an ideal BCS by week 46. The intake differences between neuter groups meant that no single model could be used to derive a TER value for feeding guidelines in female kittens through growth. There were also no significant differences between neuter groups in activity, with both showing a gradual decrease in average activity of about 25 % between 19 and 52 weeks of age (19 weeks, EN: 281 560 (95 % CI 261 244, 302 056), and CN: 289 860 (95 % CI 270 576, 309 143); 52 weeks, EN: 210 141 (95 % CI 185 934, 234 347), and CN: 218 351 (95 % CI 194 539, 242 162)).

## Discussion and conclusions

The data are consistent with dietary management being important to maintain an ideal BCS in growing female cats irrespective of age when neutered. Whilst the study is small, the controlled nature of the protocol, with regular assessment of BCS by trained panellists, provides insight into the additional impact of sexual development on weight management in the post-neuter phase. Specifically, cats that were neutered at the later time point showed a rapid post-neuter increase in food intake (also reflected in the more frequent increases in ration post-neuter; data not shown) and BW.

The data presented here support the view that earlier neutering may enable a more gradual weight gain through growth and so aid healthy weight management through growth when regulating intake to maintain an ideal BCS. It is of note that the management to an ideal BCS resulted in kittens weighing an average of 3·4 kg, similar to the average of 3·2 kg achieved by entire female cats fed *ad libitum* on a similar dry diet in the same environment^(^^11^^)^ (Fig. 1, self-regulated; middle column) and lower than the 4·1 kg average attained by the neutered kittens in that study, also neutered at 19 weeks of age and fed *ad libitum* throughout the year (Fig. 1, macronutrient profile; right-hand column). The self-regulated study^(^^11^^)^ observed that neutered cats consumed similar energy intakes to entire littermates from 37 weeks of age but continued to gain weight and percentage fat and speculated that reduced activity levels may be partly responsible for this reduced energy requirement. We measured activity in this study and saw no difference between groups at any time point.

A separate study where kittens were neutered at 26 weeks of age and allowed to eat a selection of wet foods differing in macronutrient profile *ad libitum* appeared to have a gradual weight gain and intake response (AK Hewson-Hughes, VL Hewson-Hughes, R Staunton, SJ Simpson and D Raubenheimer, unpublished results) similar to those of the EN cats (Fig. 1, TER; right-hand panel). These observations, within the context of the other studies (Fig. 1), may be used to hypothesise that kittens neutered after 26 weeks of age and fed a dry diet *ad libitum* could show more acute increases in consumption, gaining weight more rapidly and becoming overweight before reactive weight-management changes can be employed. These data could be used to hypothesise that the development of oestrogen regulation of energy intake, and its subsequent loss following gonadectomy, could be responsible for driving both the acute hyperphagia and the rapid post-neuter weight gain; further work is required to confirm this.

In addition to the TER study reported here, a parallel TER study in male kittens (also neutered at 19 weeks compared with a control group that remained entire to 31 weeks of age) observed no significant increase in mean average daily energy intake (kcal/kg BW^0·67^)^(^^17^^)^. However, following neutering at 31 weeks a significant difference in intake (CN group being greater, up to 36 (95 % CI 9, 62) kcal/kg BW^0·67^ (151 (95 % CI 38, 259) kJ/kg BW^0·67^) was observed between the two groups in weeks 34–36 and weeks 38–41, consistent with other reports in adult male cats 2·5–3·6 years of age), where an immediate and significant increase in food intake from 3 to 19 weeks post-procedure was observed^(^^18^^)^. These data may be used to hypothesise that neutering males in the early stages of sexual development may also reduce the likelihood of acute feeding behaviour changes. From a weight management and post-neuter feeding behaviour welfare perspective, neutering at or before 26 weeks irrespective of sex may be beneficial, and provides additional evidence supporting the advantages of earlier neutering alongside the social benefits and lack of evidence of health risks^(^^19^^–^^21^^)^. However, any recommendation to veterinary practices would require a critical review of all studies describing neuter-related weight gain and consideration within the context of other social and individual veterinary health concerns.
